# Occupational Physical Activity Habits of UK Office Workers: Cross-Sectional Data from the Active Buildings Study

**DOI:** 10.3390/ijerph15061214

**Published:** 2018-06-09

**Authors:** Lee Smith, Alexia Sawyer, Benjamin Gardner, Katri Seppala, Marcella Ucci, Alexi Marmot, Pippa Lally, Abi Fisher

**Affiliations:** 1 The Cambridge Centre for Sport and Exercise Sciences, Anglia Ruskin University, Cambridge CB1 1PT, UK; Lee.Smith@anglia.ac.uk; 2 Department of Behavioural Science and Health, University College London, 1-19 Torrington Place, London WC1E 6BT, UK; alexia.sawyer@ucl.ac.uk (A.S.); katri.seppala.15@alumni.ucl.ac.uk (K.S.); p.lally@ucl.ac.uk (P.L.); 3 Department of Psychology, Institute of Psychiatry, Psychology and Neuroscience, King’s College London, De Crespigny Park, London SE5 8AF, UK; benjamin.gardner@kcl.ac.uk; 4 UCL Institute for Environmental Design and Engineering, Bartlett Faculty of the Built Environment, University College London, Central House, 14 Upper Woburn Place, London WC1H 0NN, UK; m.ucci@ucl.ac.uk; 5 UCL Bartlett Faculty of the Built Environment, University College London, Gordon House, 29 Gordon Square, London, WC1H OPP, UK; a.marmot@ucl.ac.uk

**Keywords:** occupational physical activity, sedentary behaviour, stair climbing, habit, automaticity

## Abstract

Habitual behaviours are learned responses that are triggered automatically by associated environmental cues. The unvarying nature of most workplace settings makes workplace physical activity a prime candidate for a habitual behaviour, yet the role of habit strength in occupational physical activity has not been investigated. Aims of the present study were to: (i) document occupational physical activity habit strength; and (ii) investigate associations between occupational activity habit strength and occupational physical activity levels. A sample of UK office-based workers (*n* = 116; 53% female, median age 40 years, SD 10.52) was fitted with activPAL accelerometers worn for 24 h on five consecutive days, providing an objective measure of occupational step counts, stepping time, sitting time, standing time and sit-to-stand transitions. A self-report index measured the automaticity of two occupational physical activities (“being active” (e.g., walking to printers and coffee machines) and “stair climbing”). Adjusted linear regression models investigated the association between occupational activity habit strength and objectively-measured occupational step counts, stepping time, sitting time, standing time and sit-to-stand transitions. Eighty-one per cent of the sample reported habits for “being active”, and 62% reported habits for “stair climbing”. In adjusted models, reported habit strength for “being active” were positively associated with average occupational sit-to-stand transitions per hour (B = 0.340, 95% CI: 0.053 to 0.627, *p* = 0.021). “Stair climbing” habit strength was unexpectedly negatively associated with average hourly stepping time (B = −0.01, 95% CI: −0.01 to −0.00, *p* = 0.006) and average hourly occupational step count (B = −38.34, 95% CI: −72.81 to −3.88, *p* = 0.030), which may reflect that people with stronger stair-climbing habits compensate by walking fewer steps overall. Results suggest that stair-climbing and office-based occupational activity can be habitual. Interventions might fruitfully promote habitual workplace activity, although, in light of potential compensation effects, such interventions should perhaps focus on promoting moderate-intensity activity.

## 1. Introduction

Regular participation in physical activity aids the prevention of non-communicable disease and improves mental health [1]. Independent of moderate-to-vigorous physical activity, sedentary time (i.e., sitting) is also increasingly acknowledged as having a serious negative impact on health [2]. UK guidelines state that all adults (aged 19–64 years) should participate in at least 30 min of moderate-to-vigorous physical activity on at least five days a week, and should minimise extended periods of sedentary time [3]. Despite this, population levels of physical activity in the UK are low. One survey found that 26% of adults residing in England did not meet the recommended physical activity guidelines [4]. Office workers, who make up over half of the UK workforce, are at increased risk of low levels of physical activity and high sitting time. Evidence from the USA demonstrated higher levels of self-reported sedentary behaviour in office workers compared with those working in service or traditional blue-collar industries [5]. Research on office-based workers in the UK examined sedentary behaviour and physical activity during and outside working hours, using the Actigraph GT1M accelerometer [6]. Participants spent the greatest proportion of the day sedentary on work and non-work days, accounting for 68% and 60% of accelerometer wear time, respectively. Additionally, 71% of working hours were spent in sedentary activities. Parry et al. found in a sample of office workers that sedentary time, monitored by the Actical accelerometer, accounted for 81.8% of work hours (light activity 15.3% and MVPA 2.9%) [7]. In a recent Active Buildings study, Smith and colleagues found that office workers spent a large proportion of their time sitting on weekdays (10.6 h) and similarly on the weekends (10.6 h) [8].

There is a growing body of literature on interventions to promote physical activity and reduce sedentary time among office workers. The majority of interventions to increase occupational physical activity or reduce sedentary time have yielded small effects [9,10,11,12]. These interventions have typically focused on enhancing employees’ motivation to increase their activity or reduce their sitting time. For example, Malik, Blake and Suggs reported that just over half of the 58 interventions included in their review revealed a significant effect on physical activity; of all the studies included, only two included the physical environment as an intervention target [10]. This finding is supported by two further reviews conducted in the past ten years, reporting heterogeneous effects for interventions which predominantly target individual cognitions to promote physical activity [11,12]. With the aim of identifying intervention components that demonstrate promise for decreasing occupational sedentary behaviour, Gardner, Smith, Lorencatto, Hamer and Biddle’s review reported that environmental restricting, behavioural rehearsal and habit formation were only used in interventions reporting promising results [9].

However, conscious motivation to be active may not be the strongest determinant for occupational activity and sedentary time. The stable, unvarying nature of workplace environments is particularly conducive to the formation of habitual responses, which are automatically elicited when encouraging cues within those environments [13], and which can override conscious motivation in determining physical activity [14]. Workers’ habits around occupational activity and sedentary behaviour might therefore play an important part in workplace activity, but these have not yet been examined within the Active Buildings sample. Indeed, as demonstrated above, they are understudied in the wider field [9,10,11,12].

“Habitual” behaviours are those which are enacted automatically, as learned responses to associated situations [15]. Habits are learned through “context-dependent repetition” [16]. Repeated performance of an action in a specific setting reinforces a mental association between the setting and the action, to the extent that subsequently encountering the setting activates an unconscious impulse to perform the action, without conscious forethought, intention or awareness [15]. A wealth of evidence suggests that physical activity can be a habitual behaviour [14,17]. One review found a moderate to strong association between habits and self-reported physical activity, and habit and TV viewing (an archetypal sedentary behaviour) [14]. Another recent review of 52 studies concluded that the evidence revealed an association between self-reported habits and physical activity, conceptualised as any energy expenditure in leisure or non-leisure time and most frequently measured using self-report (71% of studies) [17]. However, little evidence is available around the role of habit in occupational physical activity. If workplace activity and sedentary time were to be shown to be habitual, this would suggest that workplace activity promotion interventions should focus more on promoting “good” workplace-based activity habits, and adopting strategies to tackle the automatic nature of “bad” habits (e.g., prolonged sitting). This study is a novel investigation into: (i) the extent to which people subjectively experience occupational physical activity as an automatic (i.e., habitual) action; and (ii) habit strength as a determinant of the extent of engagement in occupational physical activity and sedentary time. To our knowledge, this is the first time occupational physical activity habits were examined in an office-based population, however previous literature was drawn on to support our hypothesis [14,16,17]. It was hypothesised that occupational physical activity would be reported as a habitual behaviour, and associations would be observed between stronger self-reported habits to be active or climb stairs and increased physical activity and decreased sedentary behaviour.

## 2. Methods

Data were collected between 2013 and 2014, as part of the Active Buildings project, a cross-sectional study examining associations between office layout and stepping, standing and sitting in office-based workers (≥18 years) from 10 organisations in the UK. Full details of participant recruitment and study procedures have been reported elsewhere [18]. Participants were asked to complete the Movement at Work Survey (accessible at http://www.activebuildings.co.uk) which contained questions on standard demographics (age, sex, job role, etc.) and habit strength. Data were collected between March 2013 and March 2014. Ethical approval for the study was granted by the University College London Non-NHS Research Ethics Committee (4400/001). All participants provided informed written consent. Data were from the Active Buildings study, whose authors may be contacted through http://www.activebuildings.co.uk/. Qualified researchers can request to access the data by emailing Dr Abi Fisher at abigail.fisher@ucl.ac.uk.

Previous studies from the Active Buildings project have reported patterns of objectively-measured physical activity over weekdays and weekend days [8] and examined associations between objective physical activity and self-reported perceptions of the social and physical office environment [19]. An additional publication investigates hypothesised associations between objectively-measured physical activity and objectively-measured spatial metrics of the workplace [20]. A protocol of the project was published outlining the novel methodology and project aims. This study pertains to the secondary aim to explore socioecological correlates of occupational sedentary behaviour and physical activity [18].

### 2.1. Habit

The Movement at Work survey included the 4-item Self-Report Behavioural Automaticity Index (SRBAI) [21], a reliable and valid subscale of the Self-Report Habit Index [22], adapted to focus solely on the automaticity of habitual responses. SRBAI items followed a stem, worded in relation to two occupational physical activity domains: general workplace activity (“Being active in the workplace (e.g., walking to printers, for refreshment breaks, to coffee points) is something (e.g., I do automatically, I do without thinking)”), and stair-climbing (“Climbing stairs at work is something (e.g., I do automatically, I do without thinking)”).

Responses were recorded using a 5-point Likert scale (coded: 1 = “strongly agree”; 5 = “strongly disagree”) and was reverse-coded for analyses (1 = “strongly disagree”; 5 = “strongly agree”). Median scores were calculated to capture responses to the 4 items for “being active” and 4 items for “stair climbing”. Medians were used rather than mean averages in line with recommendations for collapsing ordinal data [23,24]. Median scores were taken to indicate the following: 1 = strong disagreement; 1.5 and 2 = disagreement; 2.5 and 3 = neutral; 3.5 and 4 = agreement; and 4.5 and 5 = strong agreement. Strong disagreement or disagreement were considered to suggest absence of habit, a neutral score (neither agree nor disagree) as neither presence nor absence of habit, agreement as a moderate habit, and strong agreement as a strong habit. 

### 2.2. The ActivPAL Accelerometer

An activity diary was developed for participants to record working patterns during the period of objective measurement. Using this diary, participants self-reported working days, time of arrival and departure from the office and removal of the monitor during the monitoring period. Days when the participant worked away from the office or did not continuously wear the monitor were excluded. Arrival and departure times were rounded-off to the nearest hour to exclude periods of commuting (rounded-down for arrival times, e.g., 08:30 to 09:00; rounded-down for departure times, e.g., 18:30 to 18:00). As hourly averages rather than sum totals were used to capture workplace behaviour, rounding-off was deemed appropriate. Missing data from arrival and departure times were inputted, using the average times from other working days of the participant where available, or working days within the participant’s organisation. Similar treatment of the physical activity data has been used elsewhere [19,20].

Participants were fitted with the activPAL3™ accelerometer (http://www.paltechnologies.com/), attached to the middle of the thigh at the workplace by trained research assistants. The device was worn all day for the following five consecutive days, capturing a minimum of 3 workdays for each participant. A waterproof adhesive dressing was fitted over the device permitting bathing and swimming without the need for removal. Participants were provided with four additional waterproof adhesive dressings in case the original dressing needed to be replaced. Previous research has shown that three consecutive days of objective data are needed to accurately measure average daily time spent in different activity intensities (e.g., [25,26]). At the end of the wear protocol, research assistants returned to the workplace to collect the devices. The activPAL has been successfully used in studies of office workers and adults [27,28] and has been validated for step count, time spent sitting, standing and walking and for identifying postural transitions [29]. Physical activity data were captured in 15-s epochs and examined by the researchers in 1-h epochs. Data were visually assessed for unusual episodes; none were identified and therefore none were excluded from analysis. For participants with ≥3 workdays of physical activity data, hourly averages of sitting, standing, stepping, step counts and sit-to-stand transitions were calculated across reported working hours. Inspection of the data suggested that they were normally distributed. Outliers (defined as values lying more than 2 standard deviations (SDs) from the mean) were removed prior to analyses.

### 2.3. Covariates

Participants self-reported their age, sex, job role (Standard Occupational Classification (SOC) code, e.g., managerial or professional) and organisation. Trained researchers collected body weight (kilograms; Tanita electronic weighing scales) and height (metres; Leicester Stadiometer) to calculate body mass index (BMI).

Participants also reported variables on the socio-cultural workplace environment, specifically “I am discouraged from leaving my desk for unscheduled breaks by the management”. Responses were recorded using a 5-point Likert scale (1 = “strongly disagree”; 5 = “strongly agree”). Discouragement of leaving one’s desk was included as a covariate, as it has previously been shown to be associated with occupational activity in this sample [19]. 

### 2.4. Analyses

Of 131 participants, a total of 116 participants had complete data available for the SRBAI, the activPAL outcomes and covariates. *T*-tests and chi-square analyses tested for socio-demographic differences between those with and without complete data. Descriptive analyses of SRBAI data were undertaken to determine self-report habit strength. Sets of unadjusted and adjusted multiple linear regression models were run to investigate the association between habit strength for “being active” and objectively measured occupational sitting, standing, stepping, step count and sit-to-stand transitions. Models for each physical activity outcome were performed separately. The same set of models was run using “stair climbing” SRBAI scores. The median score for habit strength for “being active” and “stair climbing” was used in regression models. Age, sex, job role, BMI, organisation and management discouragement of breaks were included as covariates in adjusted models. SPSS Version 24 (IBM, Armonk, New York, USA) was used for all analyses and statistical significance was set at alpha < 0.05.

## 3. Results

The majority of the sample were female (53%) with a mean age of 40 years (range 21 to 65 years). Approximately half of participants (51%) had a professional role, 23% had a managerial role, 16% had an administrative role, 3% were telephone operatives and the rest were classed as other (6%). See Table 1 for participant characteristics. There were no differences in age (*p* = 0.409), sex (*p* = 0.112), job role (*p* = 0.127) or organisation (*p* = 0.067) between participants included in analyses and those excluded due to incomplete data.

For “being active”, 81% of the sample reported having moderate or strong habits. Specifically, SRBAI median scores were: 52% strong agreement, 29% agreement, 14% neutral (neither agree nor disagree), 4% disagreement and 1% strong disagreement. Sixty-two per cent of the sample reported moderate or strong “stair climbing” habits. SRBAI median scores were: 30% strong agreement, 32% agreement, 23% neutral (neither agree nor disagree), 10% disagreement and 5% strong disagreement.

In linear regression models, adjusted for pre-specified covariates, habit strength for “being active” was associated with greater mean sit-to-stand transitions per hour (B = 0.34, 95% CI: 0.05 to 0.63, *p* < 0.05) (Table 2). In adjusted models, habit strength for “stair climbing” was negatively associated with mean occupational step count per hour (B = −38.34, 95%CI: −72.81 to −3.88, *p* < 0.05) and negatively associated with mean stepping time per hour (B = −0.01, 95%CI: −0.01 to −0.00, *p* < 0.01) (Table 3). No other associations were found.

## 4. Discussion

In this sample of 116 UK office workers, two domains of occupational physical activity were found to be habitual behaviours, with 81% of the sample reporting a habit for general activity (“being active”), and 62% reporting a habit for “stair climbing.” This finding echoes previous literature on the habitual nature of activity. For example, a systematic review and meta-analysis of the influence of habit on dietary and activity behaviours showed that physical activity and active travel were reportedly strongly habitual for many people [14,17].

Although occupational physical activity was experienced by participants as highly habitual, having a strong habit for “being active” was only associated with increased sit-to-stand transitions. The lack of association between general activity habit and other objective activity indices, such as step counts, was unexpected. In retrospect, however, when considering the nature of the occupational activities being measured, it seems sensible to assume that a habit to be active (e.g., walking to the printer, walking to break room, etc.) may not generate enough steps or standing time per se to result in a significant difference in these variables. Additionally, the observed relationship between general activity habit and sit-to-stand transitions is a promising finding, as it raises the possibility that the formation of activity habits translates into taking significantly more breaks from sitting. Due to the observational nature of the associations presented in this study, the causal determinants of this association cannot be inferred. Additional research is needed to understand whether the direction of the association and other factors which may modify the relationship, such as job type or work culture. However, recent evidence around sedentary behaviour highlights the need to pursue potential opportunities to develop interventions targeting sit-to-stand transitions. In a laboratory-controlled trial conducted over an 8 h period, interrupting sitting time every 20 min with short 2-min bouts of light-intensity or moderate-intensity walking was shown to lower postprandial glucose and insulin levels in overweight/obese adults [30]. Increasing the frequency of sit-to-stand transitions during bouts of prolonged sitting (i.e., during the working day) may be beneficial for metabolic health [31].

Surprisingly, we found stair-climbing habit strength to be *negatively* associated with occupational step count and stepping time. While effect sizes were small, this suggests that promoting habits for stair climbing may be somewhat counter-productive, leading to a net decrease in steps. These seemingly counter-intuitive findings may perhaps represent an instance of physical activity compensation, whereby an employee who climbs several flights of stairs (a form of vigorous activity that would accumulate few steps) may move less during the day than those who do not climb the stairs. Our objective activity measures did not distinguish between steps accrued by climbing stairs or by other activities, so we cannot directly assess this. Additionally, it is possible that individuals with strong habits for stair-climbing may have reported lower number of steps as the rounding of arrival/departure times from the office may exclude steps taken when entering or leaving the office using the stairs. However, evidence from previous workplace studies demonstrates the potential for people to compensate for increases in workplace activity (e.g., using a sit-stand desk) by decreasing activity and increasing their sitting time elsewhere (i.e., at home) [32].

Our findings speak to the relevance of habit to workplace physical activity, and suggest that interventions that seek to promote activity habits in the workplace have the potential to be fruitful. Habit-based activity interventions differ from non-habit-based interventions in that they focus on the promotion of activity *in stable contexts*, so that people learn associations between those contexts and engaging in physical activity, which acquire the potential to automatically trigger the activity in future [33]. Workplace interventions might therefore usefully focus on promoting the performance of certain activities—e.g., walking to the printer, or to the water-cooler—in regularly-encountered settings, such as at the same time of day, or upon finishing similar tasks, each day [34]. Future research might aim to interrogate whether office workers have habits for particular behaviours which were included in one item in the current study (e.g., walking for refreshment breaks). In addition, it would be beneficial to further examine the role of other socioecological determinants in observed associations. Unfortunately, the sample in the current study was not sufficiently large to examine differences by factors such as job role or gender. Research demonstrating differences in activity across types of employment (e.g., professional, blue collar and service occupations) could indicate that differences might also exist between job roles in office-based work [5]; future research might to test this hypothesis. In light of potential compensation effects, however, interventions might best focus more on promoting light- and moderate-intensity activities, the adoption of which increases workplace physical activity without risking declines in activity elsewhere in the day.

Limitations of our study must be acknowledged. The accuracy of self-report for capturing habit has been questioned, as habitual behaviours occur outside of awareness [35]. However, people can feasibly report, *on reflection*, that they were not aware of performing an action at the time they performed it [36]. In addition, it was not possible to precisely account for activity conducted outside of the workplace during working hours using the self-report measures used in the study. Further research could aim to capture and account for location of participant activity more precisely. A strength of this study is the objective measure of sitting, standing and stepping employed in a sample of English office-based workers. The activPAL’s inclinometer and unique positioning on the thigh allows the device to distinguish between different postures. The advantage of objective measurement of occupational activity is illustrated by recent evidence cautioning the use of self-report measures, suggesting that office-based workers tend to overestimate occupational walking, although such measures perform better for sedentary behaviour [37]. It should be noted that physical activity outcomes of the study were derived as proportions of the work hour and are therefore dependent of one another (e.g., a higher proportion of the hour spent standing displaces other activities and necessarily means that a smaller proportion of the hour is spent sitting or stepping). When interpreting the results and conclusions of this study, it is necessary to acknowledge that they are based on the assumption that independent regression models using absolute proportion values for each physical activity outcome sufficiently present the nature of these behavioural data. Alternative statistical methods using a compositional approach may have more comprehensively captured the co-dependency of the values by specifically presenting them as relative values contained within a singular whole, thereby affording more focus on the intrinsically related nature of activity outcomes than in presented analyses [38]. Finally, the relatively small sample size of office-based employees predominately residing in London limits the representativeness of the findings.

## 5. Conclusions

Results from the present study suggest that being active and climbing stairs in the workplace are subjectively experienced as habitual, and that, for “being active” at least, stronger habit predicts greater activity, in the form of increased sit-to-stand transitions. However, a negative relationship between stair-climbing habit strength and observed step count suggests that forming stair-climbing habits may be counter-productive, as people may compensate for greater stair climbing by walking fewer steps elsewhere in the workplace. Nonetheless, these findings testify to the potential for activity to become habitual in the workplace, and for habits to promote increased activity, and so better health.

## Figures and Tables

**Table 1:** Participant characteristics, objective physical activity levels and habit scores (*n* = 116).

Participants	Mean	(SD)	*n*	(%)
Age	39.64	(10.52)		
Sex				
Female			62	(53)
Male			52	(45)
Missing			2	(2)
BMI	25.82	(4.51)		
Job role				
Managerial			27	(23)
Professional			59	(51)
Administrative			19	(16)
Telephone operative			4	(3)
Other			7	(6)
Objective physical activity				
Hourly step count	440.55	(209.06)		
Proportion standing time (/h)	0.22	(0.15)		
Proportion stepping time (/h)	0.08	(0.03)		
Proportion sitting time (/h)	0.70	(0.16)		
Hourly sit-to-stand transitions	3.23	(1.21)		

**Table 2:** Median habit scores for “being active” as predictors of objective physical activity measures.

	Unadjusted Models			Adjusted Models *		
	B	95% CI	*p*	B	95% CI	*p*
Habit strength of “being active”						
Hourly step count	48.97	8.54 to 89.41	**0.018**	37.41	−3.82 to 78.64	0.075
Hourly stepping time	0.01	0.01 to 0.02	**0.025**	0.01	−0.00 to 0.01	0.128
Hourly standing time	0.03	−0.00 to 0.06	0.076	0.03	−0.01 to 0.06	0.118
Hourly sitting time	−0.03	−0.06 to −0.00	**0.031**	−0.04	−0.07 to 0.00	0.062
Hourly sit-to-stand transitions	0.22	−0.01 to 0.46	0.065	0.34	0.05 to 0.63	**0.021**

* Adjusted for age, sex, job role, BMI, organisation and management discouragement of breaks. Bold typeface indicates significance at 0.05 level.

**Table 3:** Median habit scores for “stair-climbing” as predictors of objective physical activity measures.

	Unadjusted Models			Adjusted Models *		
	B	95% CI	*p*	B	95% CI	*p*
Habit strength of “stair climbing”						
Hourly step count	−0.70	−34.75 to 33.36	0.968	−38.34	−72.81 to −3.88	**0.030**
Hourly stepping time	−0.00	−0.01 to 0.01	0.754	−0.01	−0.01 to −0.00	**0.006**
Hourly standing time	−0.01	−0.03 to 0.02	0.466	−0.01	−0.04 to 0.02	0.642
Hourly sitting time	0.01	−0.01 to 0.04	0.327	0.02	−0.02 to 0.05	0.328
Hourly sit-to-stand transitions	−0.06	−0.25 to 0.14	0.584	0.03	−0.22 to 0.28	0.827

* Adjusted for age, sex, job role, BMI, organisation, and management discouragement of breaks. Bold typeface indicates significance at 0.05 level.
